# Orchitis reveals an extragonadal primary mediastinal thymic seminoma: a coincidence or not?

**DOI:** 10.1186/s12957-017-1146-z

**Published:** 2017-04-13

**Authors:** Athanasios Tampakis, Ekaterini Christina Tampaki, Christos Damaskos, Themistoklis Feretis, Irene Thymara, Konstantinos Kontzoglou, Periklis Tomos, Gregory Kouraklis

**Affiliations:** 1 Department of Visceral Surgery, University Hospital of Basel, Spitalstrasse 21, 4056 Basel, Switzerland; 2 Second Department of Propedeutic Surgery, Laiko General Hospital, National and Kapodistrian University of Athens, Agiou Thoma 17, 11527 Athens, Greece; 3 First Department of Pathology, Laiko General Hospital, National and Kapodistrian University of Athens , 11527 Athens, Greece

**Keywords:** Thymic seminoma, Orchitis, Male germ cell tumors, Testicular intraepithelial neoplasia

## Abstract

**Background:**

Mediastinal thymic seminomas are rare male germ cell tumors with extragonadal origin that appear predominately with a cystic appearance.

**Case presentation:**

A 22-year-old male was referred to our department for further investigation of a mediastinal mass discovered incidentally during routine chest X-ray. The patient has denied any symptoms including dyspnea, chest pain, cough, fever, dysphagia, hemoptysis, weight loss, and weakness. His past medical history was remarkable for orchitis, for which he had undergone a bilateral testicular biopsy, without the latter however, indicating the presence of a germ cell tumor or a premalignant lesion. Contrast-enhanced chest computed tomography revealed a lobulated and well-marginated cystic lesion in the anterior mediastinum. Differential diagnosis included mostly a multilocular thymic cyst, a lymphoma, a seminoma, or a soft tissue tumor. Resection of the mass revealed a primary thymic seminoma.

**Conclusions:**

A surgical approach for the management of these tumors might be reasonable considering that an extensive sampling is mandatory to gain an appropriate biopsy preoperatively in order to securely confirm or refute the presence of a mediastinal extragonadal tumor. Orchitis might be a sign of a general disorder of the germ cells which might transform in time.

## Background

Male germ cell tumors with extragonadal origin (EGCTs) represent only a 2–5% of all male germ cell tumors [1]. Germ cell tumors comprise a heterogeneous group of neoplasms. Those with extragonadal origin appear along the midline, the pineal gland, mediastinum, retroperitoneum, and sacrum. Testicular germ cell tumors are classified histologically and epidemiologically into teratomas/yolk sac tumors (prepubertal), seminomas/non seminomas (postpubertal), and spermatocytic seminomas (in patients over 40 years of age) [2].

There are two major theories about the molecular biology of the pathogenesis of germ cell tumors. The first one [3] targets the transformation of the zytogene-pachytene spermatocyte. The main mechanism implicates DNA damage driven by the upregulation of p53 which provides an apoptotic trigger. In this model, an increased 12p copy number might exist.

The second one [2] suggests that under the influence of environmental factors, fetal gonocytes undergo abnormal divisions; a phenomenon called polyploidization. Under the postnatal and pubertal gonadotropin stimulation, those cells transform to invasive tumors.

Occurrence of a primary mediastinal thymic seminoma with a predominate cystic appearance has rarely been reported.

## Case presentation

**Fig. 1:**
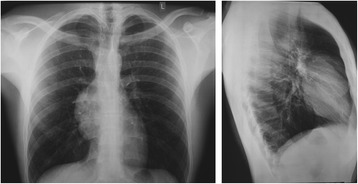
Chest X-ray shows a homogenous opacity in the *right mid upper zone* presenting no calcification

A 22-year-old male was referred to our department for further investigation of a mediastinal mass discovered incidentally during routine chest X-ray (Fig. 1). The patient has denied symptoms including dyspnea, chest pain, cough, fever, dysphagia, hemoptysis, weight loss, and weakness. Interestingly, his past medical history was remarkable for orchitis 2 years before surgery, which was treated with antibiotics. Due to an inconclusive sonogram finding related to inhomogeneous parenchym, the patient underwent a bilateral testicular biopsy, which however did not show any signs of a germ cell tumor and was negative for the presence of a premalignant lesion such as testicular intraepithelial neoplasia (TIN). The patient had no history of sexual transmitted diseases, no history of maldescended testis, and had a 5-pack-year smoking history, currently smoking one pack per day. Finally, the patient had no history of a diagnosed Klinefelter syndrome (47XXY) or myasthenia Gravis.

Clinical examination was unremarkable and did not reveal a palpable mass on the chest wall, neither has demonstrated any signs of a vena cava occlusion syndrome, while it also did not show depletion of the muscle strength regarding all muscle groups.

**Fig. 2:**
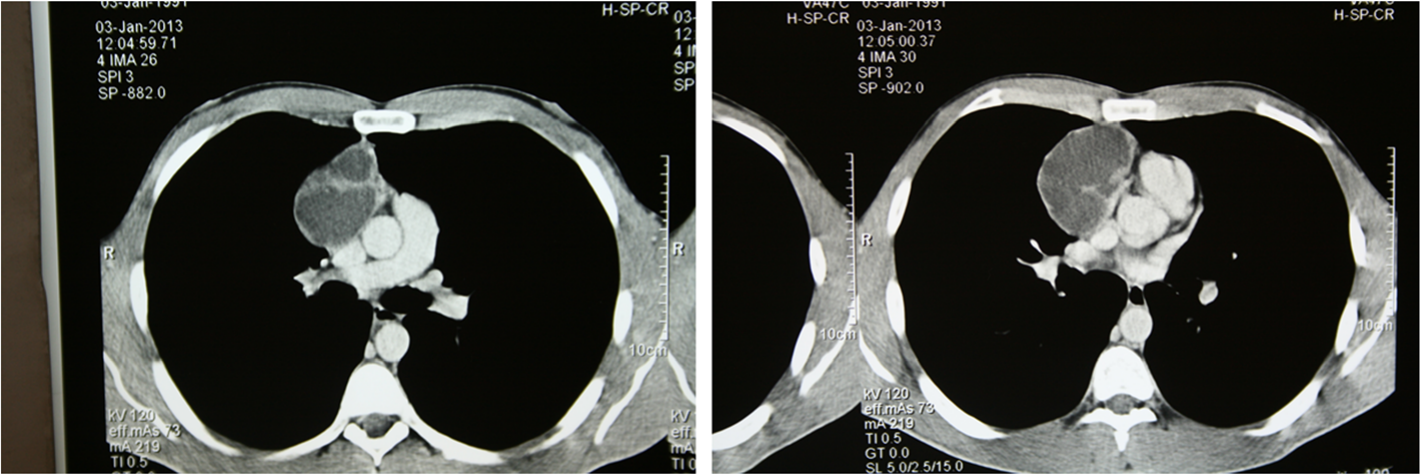
Chest CT (2013) shows an anterior mediastinal mass extending into the right side of the chest

Blood tests excluded relevant hematological disorders or signs of anemia, whereas chemistry tests revealed an elevated LDH one and a half time more than the reference rate. Alpha-fetoprotein (AFP) and human chorionic gonadotropin (hHG) values were not elevated. Contrast-enhanced chest computed tomography revealed a lobulated and well-marginated cystic lesion in the anterior mediastinum (Fig. 2). The differential diagnosis was mostly related to a multilocular thymic cyst. Speculation regarding a lymphoma, a seminoma, or a soft tissue tumor presence was highly unlikely due to their often “solid” appearance on CT. Therefore, the patient underwent a median sternotomy with an en bloc surgical excision of the tumor.

**Fig. 3:**
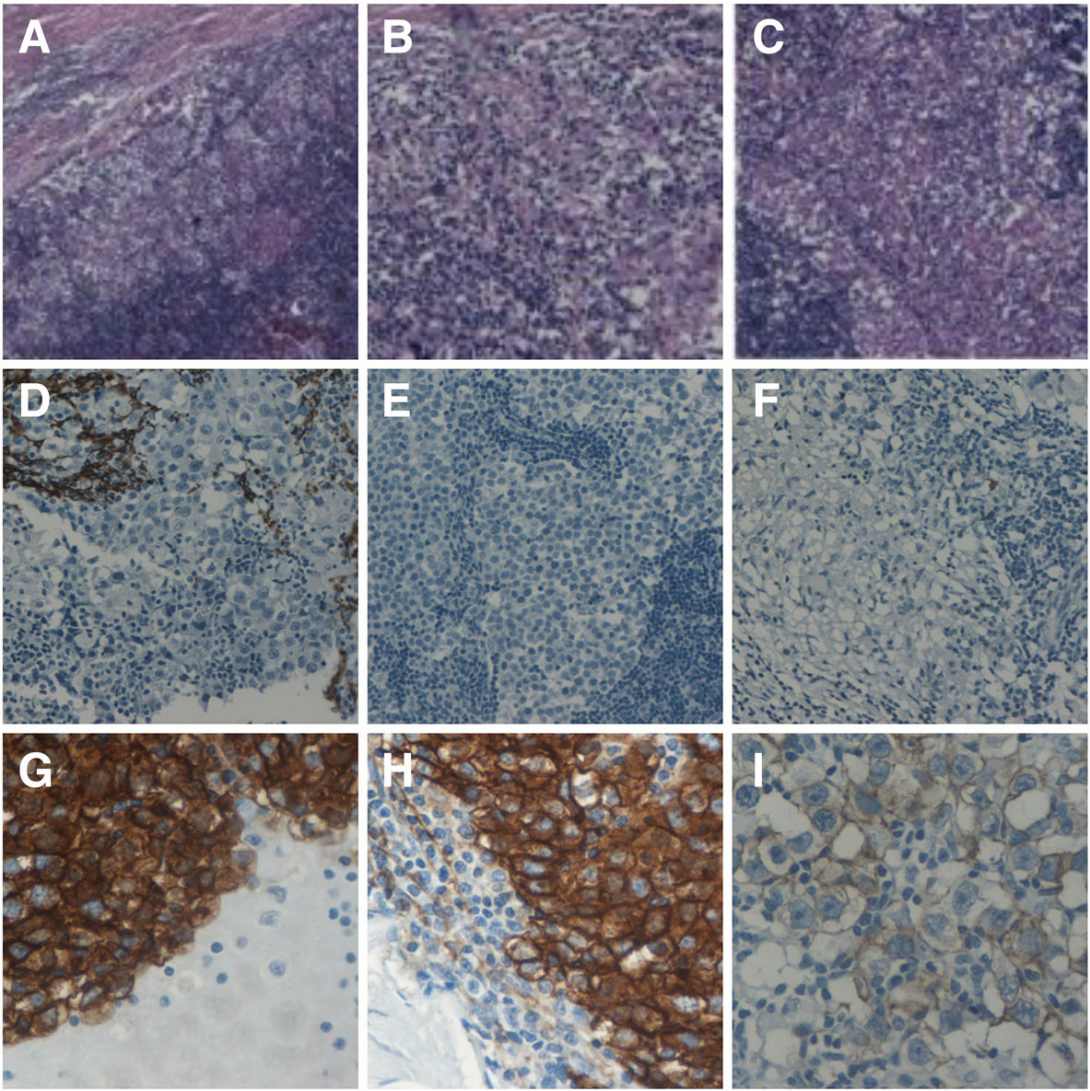
**a** Seminoma, note admixed lymphocytes (H-Ex10). **b** Seminoma showing clear cytoplasm with distinct membranes and *round* to *polygonal* nuclei with large nucleoli (H-Ex20). **c** Seminoma showing a characteristic diffuse pattern interrupted by fibrous septa with a lymphocytic infiltrate and with prominent granulomatous inflammation (H-E x5). **d** Negative stain for Pan-CK (×20). **e** Cytoplasmic membrane negative stain for SOX2 (×20). **f** Cytoplasmic membrane negative stain for CD30 (×20). **g** Cytoplasmic membrane positive stain for D2-40 (×40). **h** Cytoplasmic membrane positive stain for PLAP (×40). **i** Cytoplasmic membrane positive stain for c-kit (×40)

Surprisingly, the histopathological examination revealed a primary thymic seminoma with a predominate cystic appearance (Fig. 3). Macroscopically there appeared a solid nodule of the thymus weighting 148 g, measuring 10 × 6 × 3 cm and showing a cream-colored to pale-yellow and lobular cut surface. The tumor appeared to be well circumscribed and did not grossly involve any adjacent structures. A complete excision was performed (R0-Status) and histology showed sheets of neoplastic polygonal cells interspersed with lymphocytes consistent with pure seminoma. Histology of the neoplastic cells in more details demonstrated that the nodule of the thymus showed a diffuse arrangement of pale cells that were interrupted by fibrovascular septa containing lymphocytes and a granulomatous reaction with small clusters of epithelioid cells and giant cells of the Langhans. The tumor cells had a pale to clear cytoplasm with distinct membranes. The cytoplasmic clarity was attributable to abundant glycogen particles that were demonstrable with the periodic acid-Schiff stain. The nuclei of the tumor cells were polygonal with finely granular chromatin and flattened edges. One or more large centrally located nucleoli were present. Immunohistochemistry of the neoplastic cells showed a cytoplasmic membrane positive stain for c-kit, D2-40, and PLAP, a nuclear positive stain for oct3/4 and SALL4, and a negative stain for SOX2, Pan-CK, and CD30.

The patient received adjuvant cisplatin-based chemotherapy and 38 months after surgery he is alive and remains disease-free.

## Discussion

Two major theories for the pathogenesis of EGCTs have been described. Firstly, it has been hypothesized that these tumors are the result of a mismigration of germ cells along the urogenital ridge, which occurs during embryogenesis. Secondly, they might arise from normally distributed germ cells of the organs [1].

The cystic morphology of thymic seminoma has previously been described [4] in these rare tumors that differ morphologically from the “classic” primary mediastinal seminoma which is often illustrated as a solid mass. Histopathologically, they often tend to mimick multilocular thymic cysts [5]. The seminomatous component grows typically along the cystic walls of the tumor [6] and therefore an extensive sampling is mandatory in order to determine whether a seminoma exists. The latter suggests that a surgical approach of these tumors is warranted in the context of possible difficulties to gain an appropriate biopsy sample preoperatively in order to provide a secure diagnosis.

According to the International Germ Cell Cancer Collaborative Group (IGCCCG), the treatment of extragonadal seminomas with good or intermediate prognosis is based on chemotherapy [7]. The role of chemotherapy and/or radiotherapy along with additional surgery if the mass is still illustrated after first line treatment has also been confirmed in 2008 in the European Consensus conference for the management of germ cell cancer [8].

Interestingly, one third of the patients with EGCT may harbor TIN in clinically normal testicles. The risk of developing metachronous testicular cancer 10 years after treatment is 6.2% for tumors with a primary mediastinal location. However, due to the fact that the vast majority of these patients receive a platin-based chemotherapy, which might eliminate TIN, the standard use of testicular biopsies after treatment in the follow-up period is not recommended according to the European consensus guidelines [8].

Although the presence of orchitis might reveal a germ cell tumor of the testicles, our patient had negative bilateral biopsies but still developed an EGCT. In the study of Bokemeyer and colleagues [1] regarding 635 extagonadal seminomas (341 primary mediastinal seminomas), a testicular biopsy has been performed in 71 patients (60 patients with a retroperitoneal seminoma, and 11 patients with a mediastinal seminoma) and interestingly, atrophic or fibrotic testis was found in 22 patients, a Sertoli cell syndrome in 2 patients, and TIN in 6 patients. The same study demonstrated “unspecific” changes in around half of the cases, while in around 10% of the cases confirmed the presence of TIN with a diagnosed EGCT. However, a correlation of EGCT development with these changes is difficult to support. Nevertheless, the question raised here would be whether orchitis could possibly be a sign of a disorder of the germ cells which might at some point follow a malignant transformation. If the latter could be confirmed, then a diagnostic evaluation regarding the presence of an extragonadal germ cell tumor in patients where testicular biopsy is mandatory might be warranted. In this case and according to the most common locations where such a tumor could grow, a minimum of chest X-Ray combined with abdominal sonogram might be warranted.

## Conclusions

A surgical approach for the management of these tumors might be reasonable considering the fact that extensive sampling is mandatory to gain preoperatively an appropriate biopsy sample in order to securely confirm or refute the presence of a mediastinal extragonadal tumor. Orchitis might be a sign of a general disorder of the germ cells which might transform in time.

## Abbreviations

ECGTs: Male germ cell tumors with extragonadal origin
TIN: Testicular intraepithelial neoplasia
